# High corrosion resistance and weak corrosion anisotropy of an as-rolled Mg-3Al-1Zn (in wt.%) alloy with strong crystallographic texture

**DOI:** 10.1038/s41598-017-16351-z

**Published:** 2017-11-22

**Authors:** B. J. Wang, D. K. Xu, Y. C. Xin, L. Y. Sheng, E. H. Han

**Affiliations:** 1 School of Environmental and Chemical Engineering, Shenyang Ligong University, Shenyang, 110159 China; 2 CAS Key Laboratory of Nuclear Materials and Safety Assessment, Institute of Metal Research, Chinese Academy of Sciences, Shenyang, 110016 China; 3 Chongqing University, College of Materials Science & Engineering, Chongqing, 400030 China; 4 Peking University, Shenzhen Institute, Shenzhen Key Lab Human Tissue Regenerate & Repair, Shenzhen, 518057 China

## Abstract

Combining with electrochemical corrosion measurements, immersion and hydrogen evolution testing performed in 0.9 wt.% NaCl solution at 37 °C, the corrosion resistance of an as-rolled Mg-3%Al-1%Zn alloy before and after a 3% compressive strain along the rolling direction was investigated. Results revealed that the corrosion behavior of differently oriented surfaces of the as-rolled samples with a strong basal texture was obviously different. Among them, the corrosion rate of sample surface with the orientation parallel to the normal direction (ND) of the plate was the fastest, the corrosion rate of sample surface with the orientation parallel to the rolling direction (RD) of the plate took the second place and the corrosion rate of sample surface with the orientation parallel to the transverse direction (TD) was the slowest. After being pre-strained, the activation of a high density of {10–12} twins could remarkably reduce the corrosion rate of surrounding α-Mg matrix and simultaneously weaken the corrosion anisotropy between differently oriented samples. The main reason was that similar to grain boundaries, twin boundaries acted as physical barriers to the corrosion attack. Moreover, the activated twins increased the protectiveness of surface films and then suppressed the micro corrosion couples occurred in twinned grains.

## Introduction

Due to the good biocompatiablity and biodegradation, Mg alloys are the most promising candidates for the applications being as the biodegradable implant materials^1^. Although the applications of Mg alloys implants could possibly avoid the secondary operation after the injury being healed^2–5^, the mechanical degradation due to their high corrosion rates can hardly meet the strength requirement under some situations such as stents for blood vessels, screws and plates for fixing hard tissues^6^. Thus, this drawback greatly limits their applications as implant materials in the real medical practices. To control the corrosion behavior of Mg alloys, researchers have proposed several approaches such as adjusting alloy composition^7^, purification^8,9^, optimizing the microstructure^10–15^ and surface coatings^16–18^.

In addition to such methods, researchers found that the controlling of crystallographic texture can also remarkably influence the corrosion resistance of Mg alloys^19–25^. Generally, a densely packed crystallographic plane (i.e. {0002} basal plane) exhibits a slower corrosion rate than a loosely packed plane due to its higher atomic coordination and stronger atomic bonding^6^. Song *et al*. reported that the corrosion rate of a cross-sectional surface being mainly composed of {10–10} and {11–20} prism planes was about 8.42 times higher than that of a rolling surface consisting of {0002} basal planes^19^. Moreover, the film formed on the basal planes of pure Mg grains is more protective than that on non-basal planes^26^. Since strong crystallographic texture in Mg alloys can easily be formed during severe plastic deformation (such as rolling^19,21^ and extrusion processes^27,28^), the corrosion anisotropy in as-rolled and as-extruded AZ31 Mg alloys^19–22,27,28^, magnesium single crystals^24^ and pure Mg^25^ will be unavoidable. Although the previously reported approaches are helpful for improving the corrosion resistance of Mg alloys such as AZ31 and AM50, they can hardly eliminate or weaken the corrosion anisotropy between differently oriented surfaces.

Generally, {10–12} <10–11> extension twinning can be widely occurred during severe plastic deformation in wrought Mg alloys^28,29^. Since the twin boundary (TB) is a special kind of coherent high-angle grain boundary (GB) with the lowest interfacial energy^30^, the occurrence of twinning should influence the corrosion resistance of Mg alloys. Owing to its complex and dynamic formation process, the effect of twinning on corrosion properties is very complicated^29^. To date, the influence of twins on corrosion properties of magnesium alloys is still in debate. For example, Aung *et al*. reported that the existence of twins could accelerate the corrosion of an as-rolled AZ31 sheet^31^. However, Zou *et al*. reported that the extension twinning could accelerate the formation of homogeneous oxide film in twinned area and increase charge transfer resistance, resulting in the enhanced corrosion resistance of Mg-Y alloys^29^. Moreover, the activation of the {10–12} <10–11> twins can lead to a reorientation of 86.3° of the crystal lattice with respect to the untwinned Mg matrix^32–35^. Therefore, it can be predicted that the wide activation of twins could remarkably weaken the intensity of basal texture and then influence the corrosion anisotropy between different oriented surfaces. Moreover, the wide activation of twins can refine the grain structure owing to the formation of twin boundaries in the interior of grains. Since grain boundaries could act as physical corrosion barriers in Mg alloys, the corrosion rate of fine-grained microstructure is lower than that of the coarse-grained microstructure^31^. Similar to grain boundaries, twin boundaries (TBs) should also be the physical barriers to the corrosion attack. Based on the description mentioned above, the formation of high densities of twins could be considered as an effective way for improving corrosion resistance and weakening the corrosion anisotropy of wrought Mg alloys. However, so far, no relevant work can be referred. In this work, the target is to investigate and compare the corrosion anisotropy of an as-rolled Mg-3%Al-1%Zn alloy containing with and without high densities of twins. Additionally, the underneath mechanism about the effect of activated twins on corrosion behavior of Mg alloys will be deeply discussed.

## Results and Discussion

### Microstructural characterization

**Figure 1:**
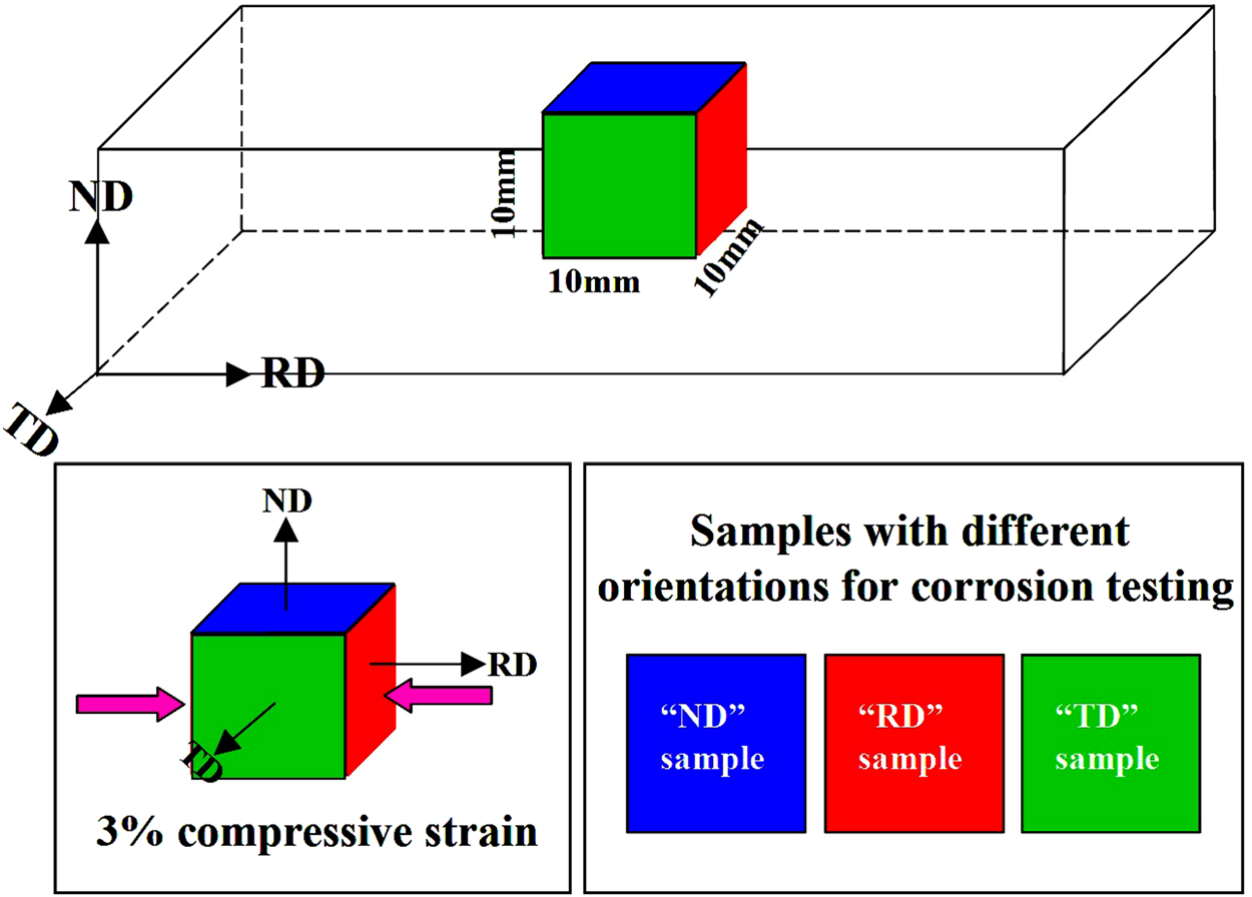
Schematic illustration of differently oriented samples for corrosion testing. Here, orientations of “ND”, “RD” and “TD” in the samples were defined as normal, rolling and transverse directions of the rolled plate, respectively.

Figure 1 depicts the dimensions and orientations of “ND”, “RD” and “TD” samples cut from the as-rolled AZ31 Mg alloy plate.

**Figure 2:**
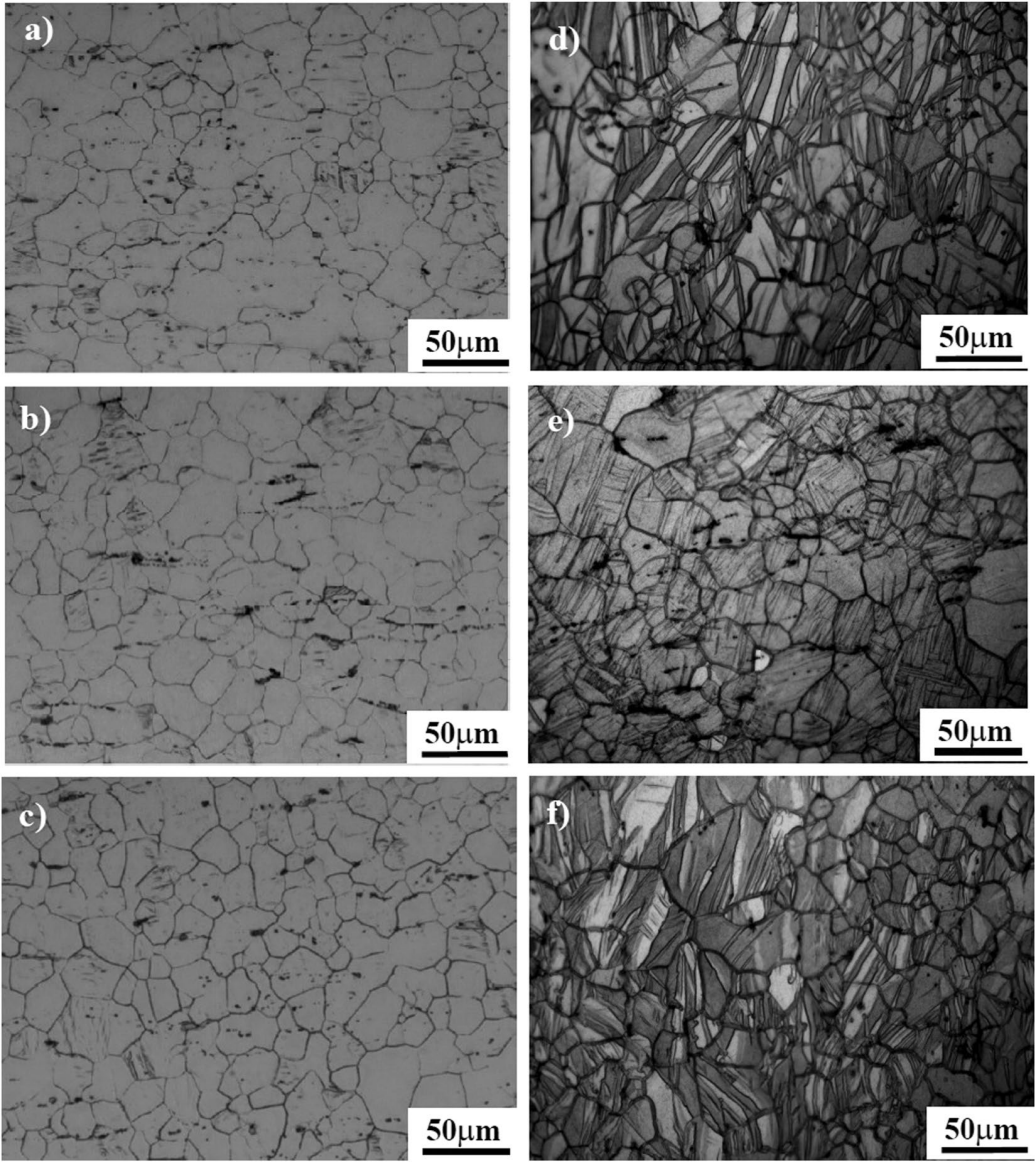
Optical observations to the microstructure of: (**a**) ND, (**b**) RD and (**c**) TD samples with no compressive strain; (**d**) ND, (**e**) RD and (**f**) TD samples after a 3% compressive strain along the RD direction.

The metallurgical microstructures of three oriented samples before and after a 3% compressive strain along the rolling direction are shown in Fig. 2.

**Figure 3:**
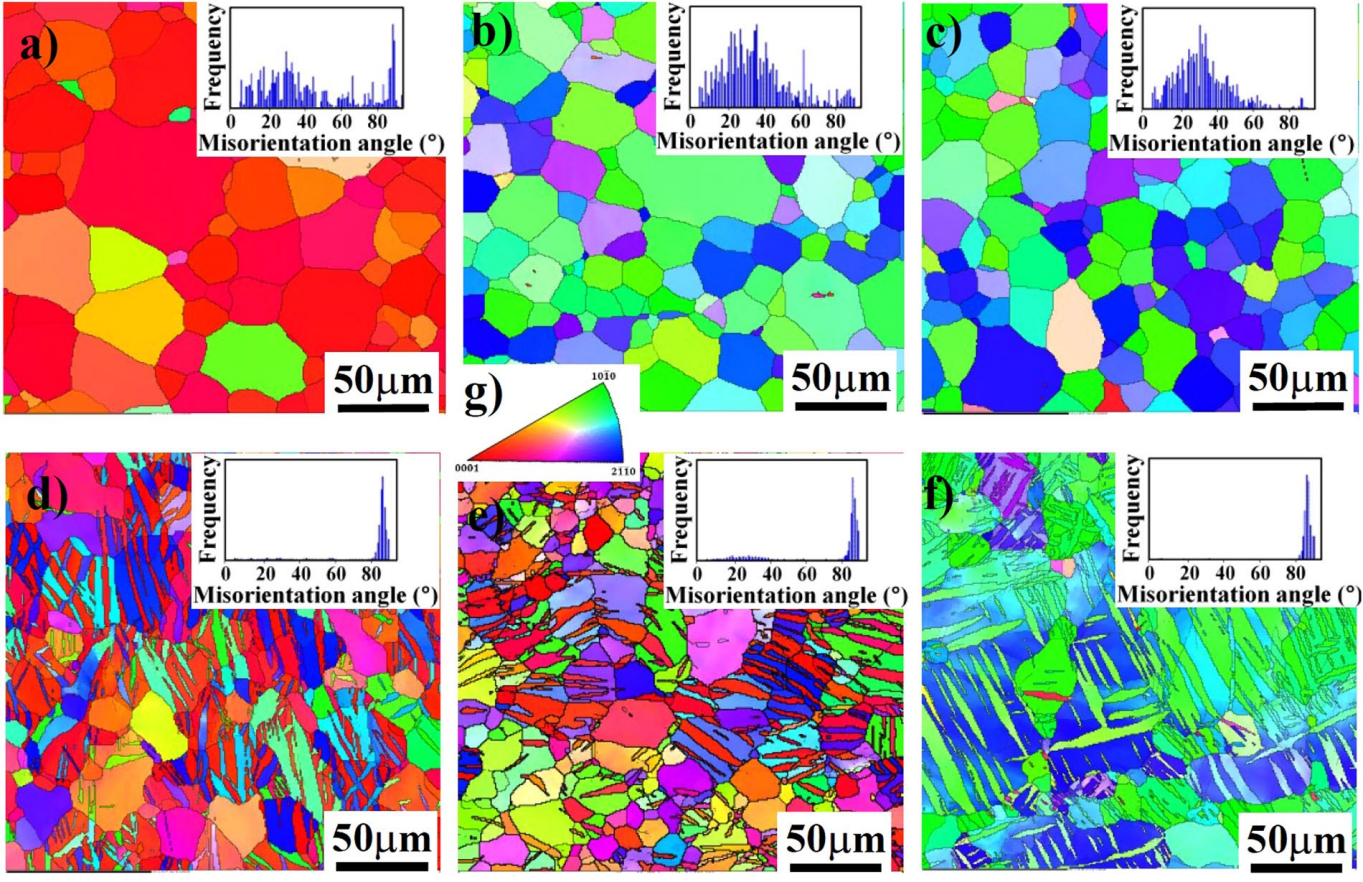
EBSD analyses to differently oriented samples. Images (**a**, **b** and **c**) and (**d**, **e** and **f**) are the orientation maps to the grain structure of the “ND”, “RD” and “TD” samples before and after a 3% compressive strain along the RD direction. Image (**g**) is the stereographic triangle and the colors correspond to the crystallographic axes of the grains. The insets are the distribution of misorientation angle between grain boundaries for different samples.

It reveals that for the as-rolled condition, the grain sizes of “ND”, “RD” and “TD” samples are almost the same and their average sizes are 40 μm. Meanwhile, a little of twins (with a volume fraction of less than 5%) can be observed on surfaces of three differently oriented samples, which could be induced by the thermomechanical process and even mechanical polishing^36^. After being pre-strained, twins were widely activated in differently oriented samples and their volume fraction occupied about 60% of the whole matrix. Considering the introduced high densities of twin boundaries (TBs), the grain structures of differently oriented samples were remarkably refined and lamellar-structured twins were widely formed in the interior of grains. To further confirm the type and misorientation distribution between twinned and untwinned areas, EBSD analyses of three differently oriented samples before and after a 3% compressive strain were performed, as shown in Fig. 3.

**Figure 4:**
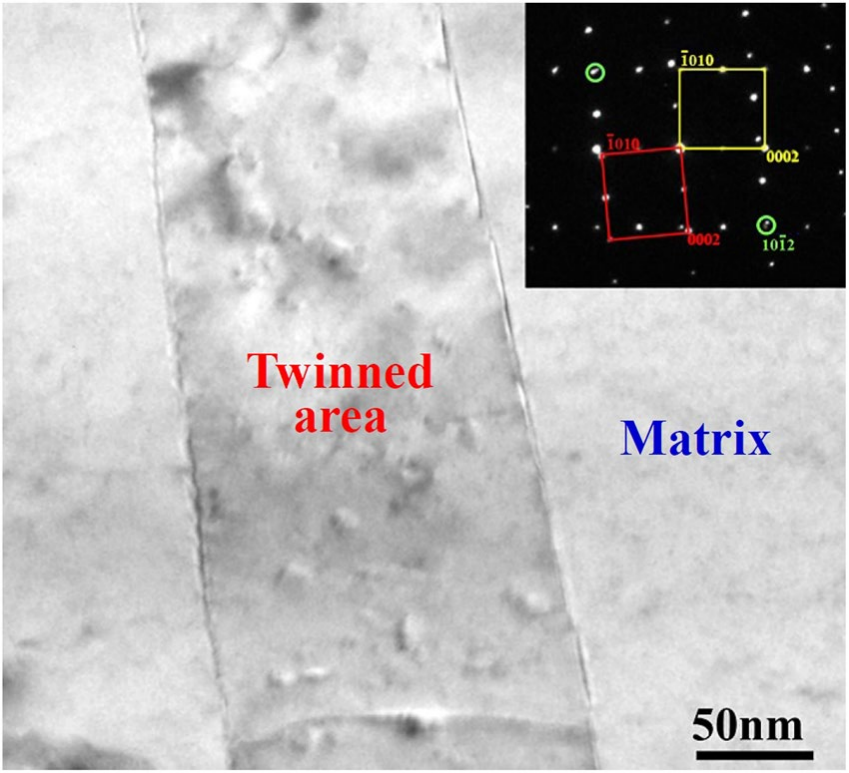
TEM result showing the morphology of activated {10–12} twins. The inset is the corresponding selected area diffraction pattern of the twined area.

**Figure 5:**
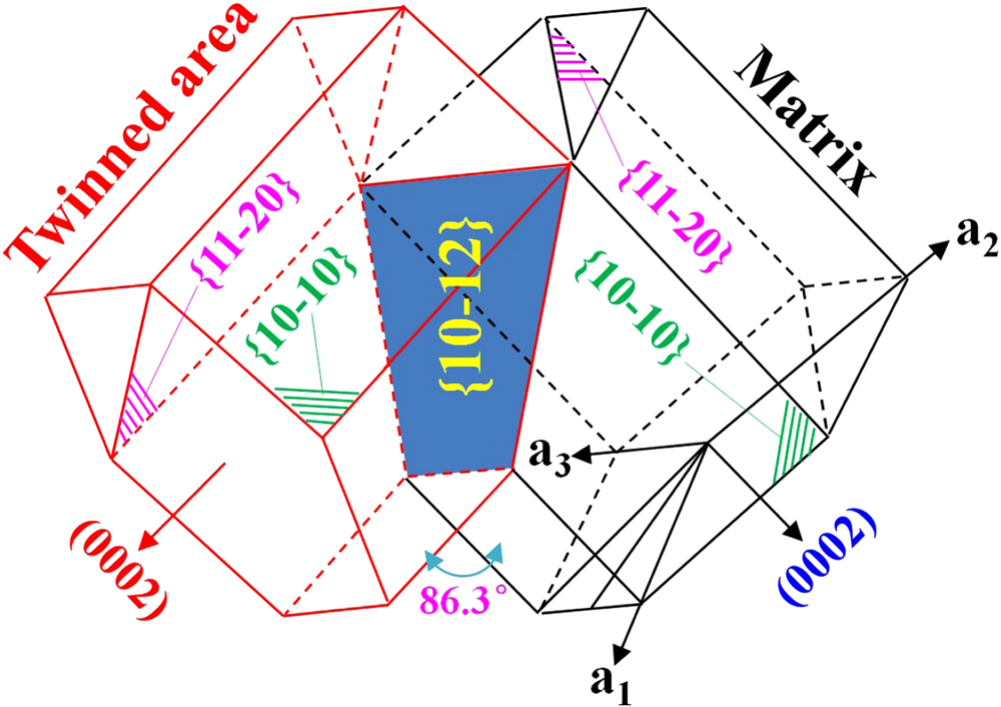
A sketch showing the crystallographic orientation of {10–10} and {11–20} prism planes in the matrix and twinned area with respect to the HCP unit cell.

Since the peak of misorientation angles concentrates at 86.3° (insets in Fig. 3(d–f)), the type of activated twins in compressive strained samples is the {10–12} extension twinning, which is further confirmed by transmission electron microscopy (TEM) result, as shown in Fig. 4. After twinning, the crystallographic orientation of {10–10} and {11–20} prism planes in the twinned area will accordingly rotate with respect to those in the matrix, as illustrated in Fig. 5. Based on the colors in the stereographic triangle (Fig. 3(g)), it reveals that for the “ND” samples with no compressive strain, the exposed crystallographic planes of most grains are {0002} basal planes (Fig. 3(a)). After compression along the rolling direction, the exposed crystallographic planes in twinned areas are {10–10} and {11–20} prism planes (Fig. 3(d)). For the “RD” samples with no compressive strain, the exposed crystallographic planes of most grains are {10–10} and {11–20} prism planes (Fig. 3(b)). After being pre-strained along the rolling direction, the exposed crystallographic planes in twinned areas are mainly {0002} basal planes (Fig. 3(e)). For the “TD” samples before and after compressive strain, the exposed crystallographic planes in twinned and untwinnned areas are all the {10–10} and {11–20} prism planes (Fig. 3(c),(f)). Previous work demonstrated that due to the 6-fold symmetry of the hexagonal close packed (HCP) structure, the orientation requirement of (10–12), (−1012), (1–102), (−1102), (01–12) and (0–112) twin planes with respect to the stress direction for the activation of {10–12} twinning is the same^37^. Therefore, the activation of {10–12} twins needs a favorable orientation to cause the movement of atoms along twin planes. Since the compression direction is parrallel to the rolling direction, the activation of {10–12} twins will cause a 86.3° rotation of c-axis in the twinned areas towards the rolling direction. Follwing this, it can easily explain the crystallographic orientation of planes in twinned areas of three differently oriented samples after compressive strain. Meanwhile, for Mg alloys containing {10–12} twins, the detwinning might take place under certain conditions such as reversed loading^38–40^, strain path changed loading^41^ and annealing treatment^42^. Since the detwinning is the migration process of twin boundary and does not need the nucleation of new twins, the activation stress for detwinning is often much lower than that of twinning nucleation. Based on previous work mentioned above, it can be predicted that the widely activated {10–12} twins in pre-strained samples are instable and the occurrence of detwinning phenomenon is closely dependent on the subsequent loading and heat treatment.

**Figure 6:**
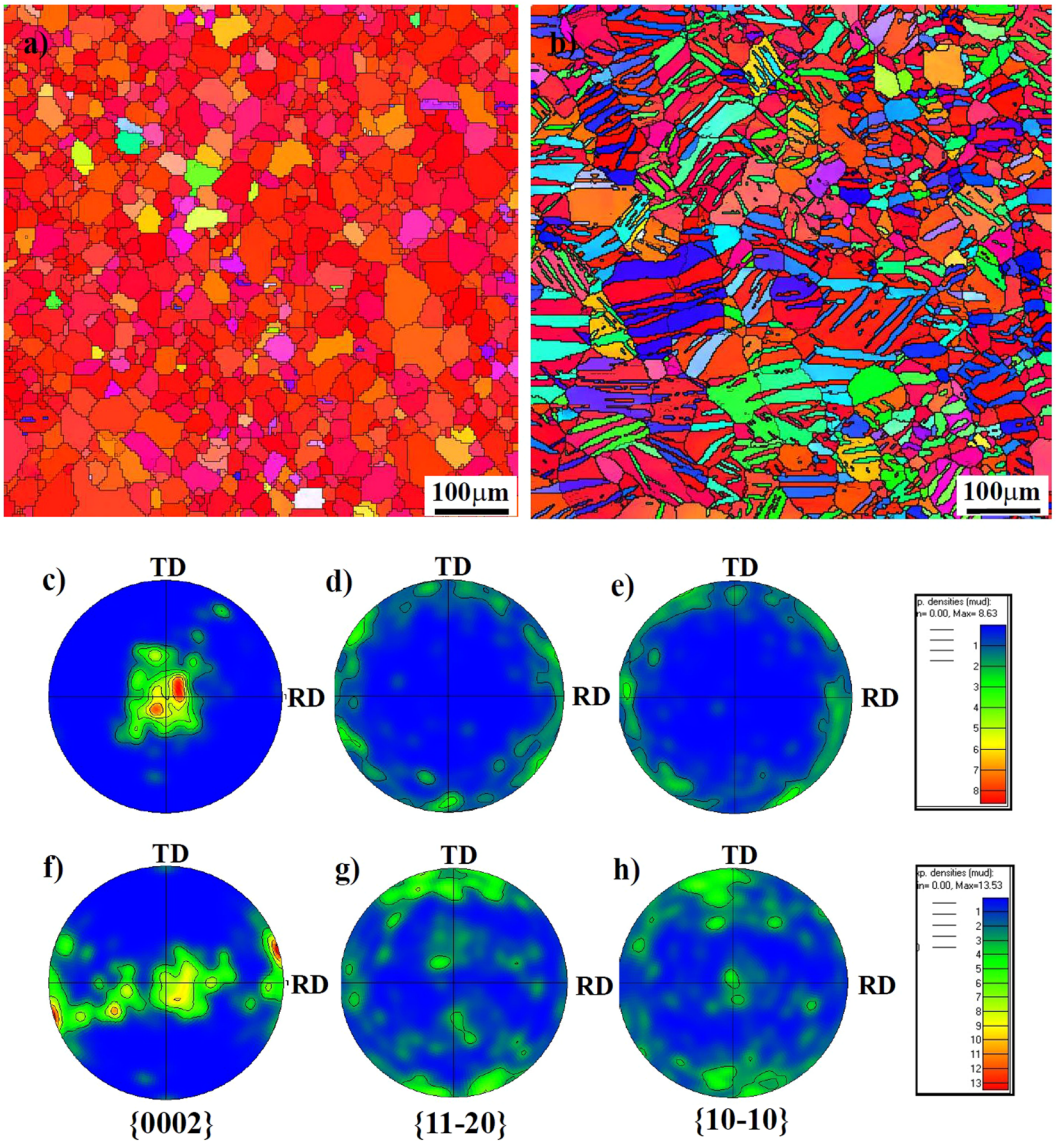
Micro texture analysis from EBSD. Images (**a**) and (**b**) are the orientation maps to the grain structure of the “ND” samples before and after a 3% compressive strain along the RD direction. Images (**c**–**e**) and (**f**–**h**) are the (0002), (11–20) and (10–10) pole figures of non-strained and pre-strained “ND” samples, respectively.

To reflect the effect of the activation of {10–12} twins, the crystallographic textures of “ND” samples were selected for analysis, as shown in Fig. 6. Based on the {0002} basal figures before and after being pre-strained (Fig. 6(c,f)), it reveals that the activated {10–12} twins could weaken the original basal texture formed in the as-rolled plate. Moreover, it further confirms that the c-axis of twinned areas is parrallel to the rolling direction of the plate. However, the variations of (10–10) and (11–20) pole figures before and after compression indicate that the crystallographic orientation distribution of prism planes is quite random and the activation of {10–12} twins can hardly influence their distribution (Fig. 6(d,g,e,h)).

**Figure 7:**
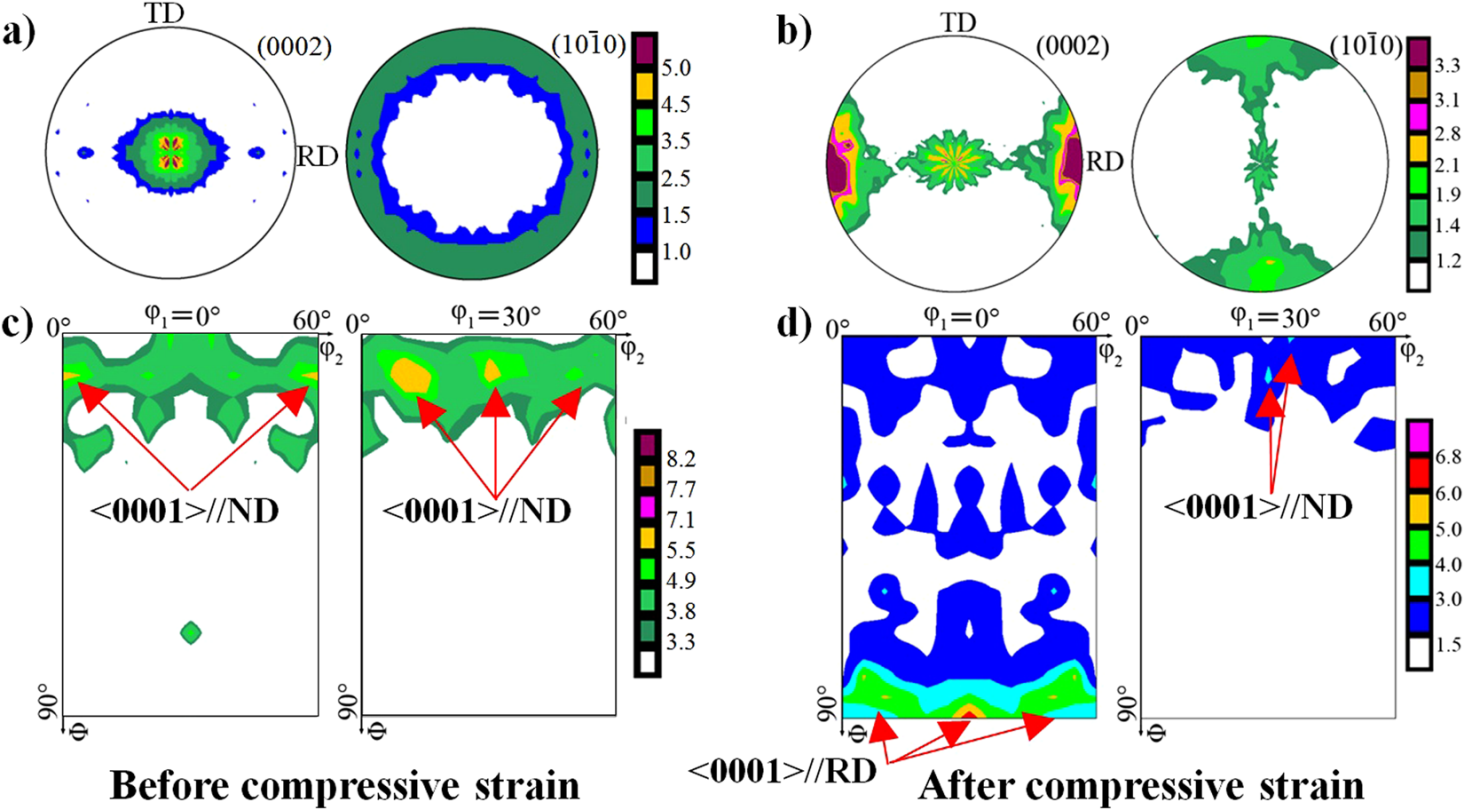
Macro texture analysis from XRD: (**a**) and (**b**) (0002) and (10–10) pole figures, (**c**) and (**d**) ODF sections at φ_2_ = 0° and φ_2_ = 30° of the “ND” samples before and after a 3% compressive strain.

Figure 7 shows the measured pole figures and the calculated orientation distribution function (ODF) maps of the “ND” sample before and after a 3% compressive strain by using X-ray diffractometer (XRD). On the basis of the measured pole figures and ODF maps, it can be seen that only one texture component (i.e.<0001>//ND) exists in the unstrained sample (Fig. 7(a),(c)), whereas two texture components (i.e. <0001>//ND and <0001>//RD) are present in the pre-strained sample (Fig. 7(b),(d)), which are in good agreement with those from EBSD.

### Immersion and hydrogen evolution testing

**Figure 8:**
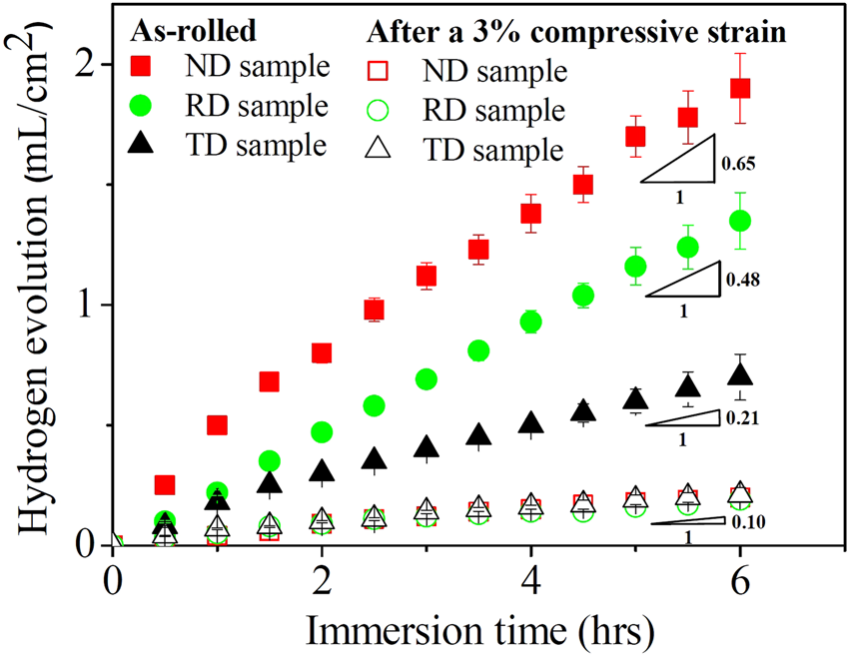
Hydrogen evolution curves of the non-strained and pre-strained “ND”, “RD” and “TD” samples at the OCP in 0.9 wt.% NaCl solution at 37 °C.

Figure 8 shows the variation curves of hydrogen evolution versus immersion time for the “ND”, “RD” and “TD” samples before and after a 3% compressive strain along the RD direction. It can be seen that for the as-rolled plate, obvious difference in hydrogen evolution rates of differently oriented samples can be observed. Based on the slopes of hydrogen evolution curves, it reveals that the hydrogen evolution rate of “ND” samples is about 1.4 and 3.1 times as high as those of “RD” and “TD” samples, respectively. After being pre-strained, the hydrogen evolution rates of three differently oriented samples were remarkably reduced and no difference in their hydrogen evolution rates was observed. Generally, the hydrogen evolution rate from a corroding Mg alloy at its open-circuit potential (OCP) reflects the electrochemical activity to some degree^19^. Therefore, it demonstrates that the activation of high densities of {10–12} twins could improve the corrosion resistance of differently oriented samples and weaken their corrosion anisotropy.

**Figure 9:**
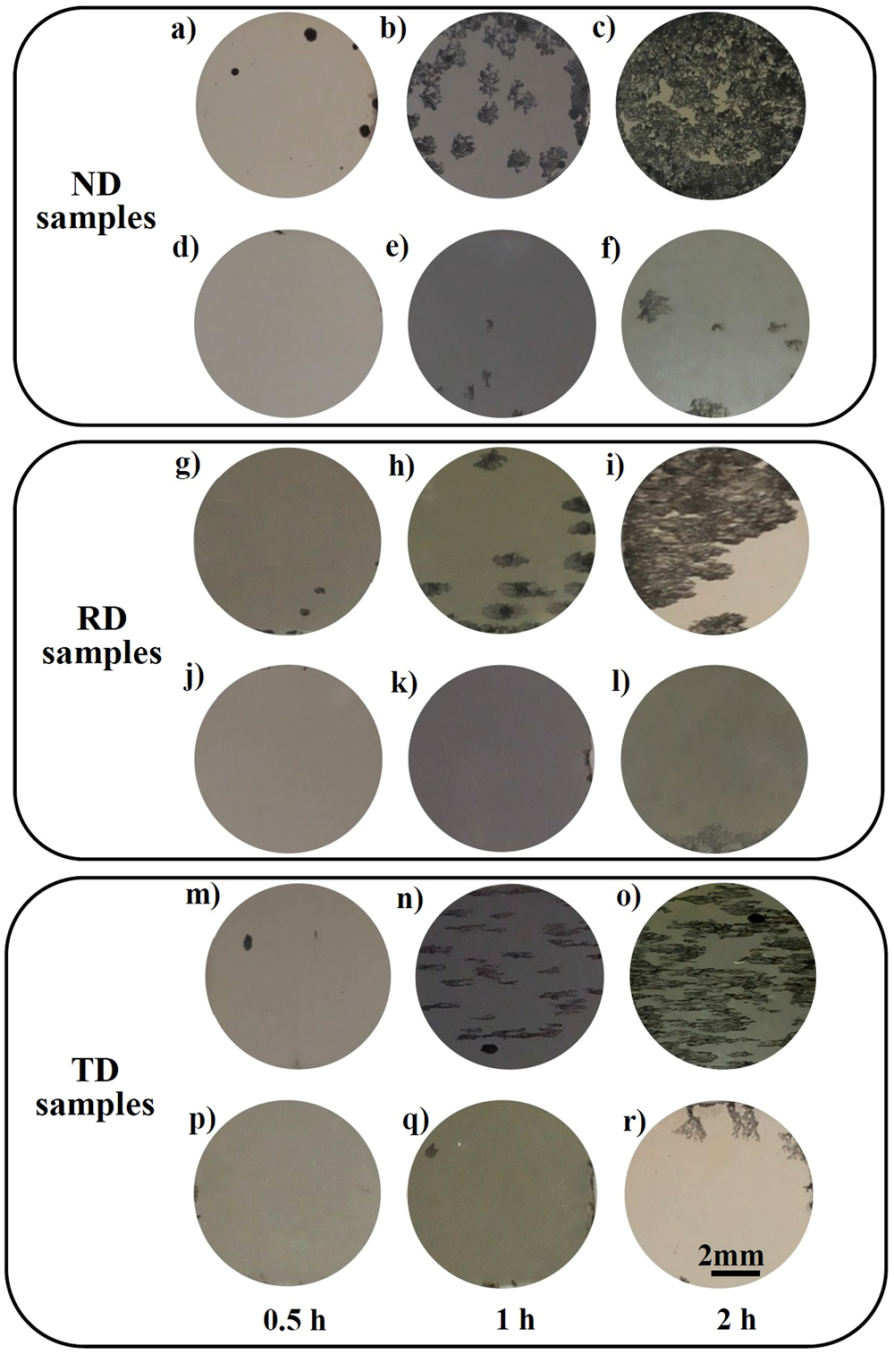
Macro corrosion morphologies after being immersion in 0.9 wt.% NaCl solution at 37 °C. Images (**a b** and **c**), (**g h** and **i**) and (**m n** and **o**) are the observations to corroded surfaces of the non-strained “ND”, “RD” and “TD” samples. Images (**d e** and **f**), (**j k** and **l**) and (**p q** and **r**) are the observations to corroded surfaces of the pre-strained “ND”, “RD” and “TD” samples.

To confirm it, the corroded surfaces of different samples after hydrogen evolution testing for 0.5 h, 1 h and 2 h were observed, as shown in Fig. 9.

For the as-rolled condition, the corrosion attack on differently oriented surfaces can be obviously observed. Meanwhile, the ranking sequence on the basis of their corrosion severity is “ND”, “RD” and “TD” samples, which is consistent with the trend in hydrogen evolution curves. After being pre-strained, the corrosion attack on differently oriented surfaces becomes much weak and only a few of localized corrosion can be observed even after immersion for 2 h.

### Electrochemically-evaluated corrosion response

**Figure 10:**
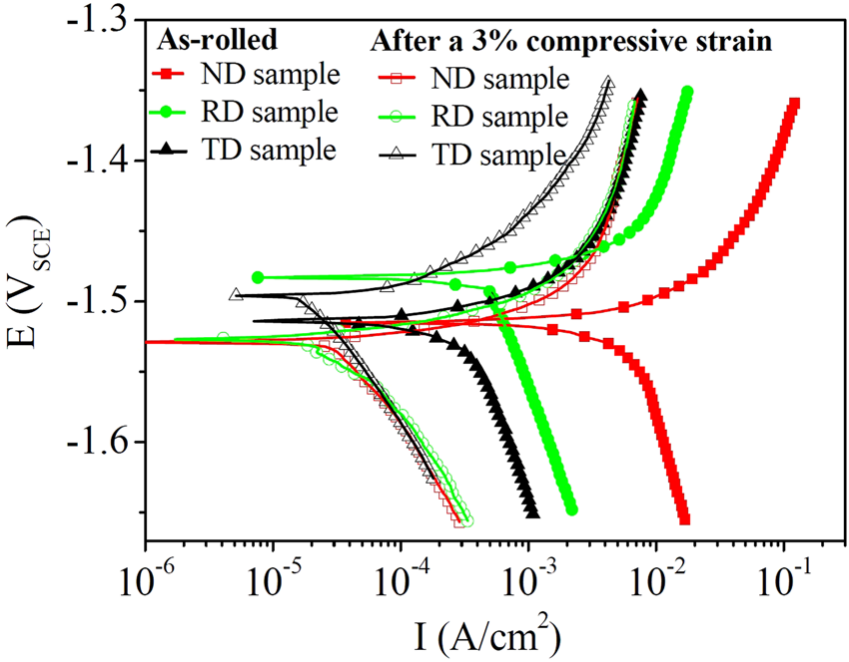
Potentiodynamic polarization curves of differently oriented samples before and after a 3% compressive strain along the RD direction.

Figure 10 shows the potentiodynamic polarization curves of the non-strained and pre-strained “ND”, “RD” and “TD” samples. It can be seen that for all the samples, the anodic and cathodic branches of the measured polarization curves are not symmetrical and the increase rate of current density in anodic branches is much greater than that in the cathodic branches. Similar phenomenon in other Mg alloys such as ZE41 and AZ91 has also been reported in the literatures^43,44^. Since the anodic branches were not suitable for fitting analysis due to the influence of pitting corrosion^43^, the corrosion parameters were determined from the cathodic branches of polarization curves by Tafel extrapolation, as listed in Table 1. It can be seen that for the as-rolled condition, the “ND” samples present the lowest corrosion current density (i_corr_) of 5.2 × 10^−3^ A/cm^2^, whilst i_*corr*_ values of “RD” and “TD” samples are about 4.6 × 10^−4^ and 2.7 × 10^−4^ A/cm^2^, respectively. Thus, the corrosion anisotropy between differently oriented samples is quite obvious. After being pre-strained, the corrosion rates of three differently oriented samples decreased at least 10 times lower than those of as-rolled samples. Moreover, the difference in corrosion rates between differently oriented samples is slight and the i_*corr*_ values of “ND”, “RD” and “TD” samples are 2.7 × 10^−5^, 2.0 × 10^−5^ and 2.2 × 10^−5^ A/cm^2^, respectively.

**Table 1:** Fitting results of the polarization curves of differently oriented samples before and after a 3% compressive strain along the rolling direction.

Condition		*i*_*corr*_ (A/cm^2^)	*E*_*corr*_ (V_SCE_)
As-rolled	“ND”	(5.2 ± 0.5) × 10^−3^	−1.515 ± 0.005
	“RD”	(4.6 ± 0.2) × 10^−4^	−1.482 ± 0.007
	“TD”	(2.7 ± 0.3) × 10^−4^	−1.515 ± 0.004
After a 3% compressive strain	“ND”	(2.7 ± 0.2) × 10^−5^	−1.525 ± 0.007
	“RD”	(2.0 ± 0.3) × 10^−5^	−1.530 ± 0.007
	“TD”	(2.2 ± 0.2) × 10^−5^	−1.495 ± 0.004

**Figure 11:**
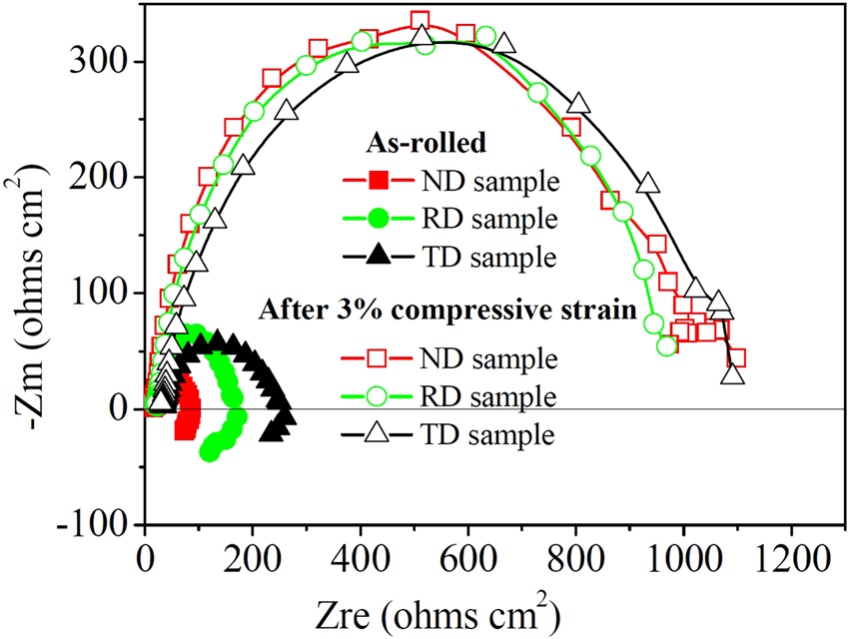
Electrochemical impedance spectra of differently oriented samples before and after a 3% compressive strain along the RD direction, respectively.

To further elucidate the corrosion mechanism of differently oriented samples before and after a 3% compressive strain, electrochemical impedance spectroscopy (EIS) was performed and presented in the form of Nyquist plots, as shown in Fig. 11. It can be seen that the Nyquist plots of samples with and without compressive strain have different features. In the case of the as-rolled condition, the Nyquist plot consists of one capacitive loop at high frequency region and one partially resolved inductive loop at low frequency region. For the pre-strained samples, only capacitive loops at high frequency are observed in the Nyquist plots and their sizes are basically the same. Meanwhile, the capacitance loops of compressed samples are relatively larger, indicating their higher corrosion resistance and better corrosion protection of the formed surface films. On the contrary, the sizes of capacitance loops for the as-rolled samples are relatively smaller and different with each other, indicating their lower and anisotropic corrosion resistance. Previous studies indicated that the existence of the low frequency inductive loop was due to the initiation of localized corrosion^45,46^. Song *et al*. proposed that the presence of Cl^−^ anions in the corrosive medium made the surface film more active or vulnerable to the delaminated area within the film^47^. Thus, the existence of low frequency inductive loops for the as-rolled samples was attributed to the insufficient protection of their surface films.

**Figure 12:**
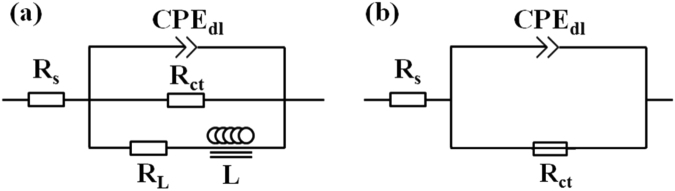
Equivalent circuit models used for fitting the impedance spectra of: (**a**) and (**b**) the “ND”, “RD” and “TD” samples before and after a 3% compressive strain along the RD direction, respectively.

Figure 12 shows the electrochemical equivalent circuits for fitting the EIS spectra of samples before and after a 3% compressive strain. In the equivalent circuit for as-rolled samples (Fig. 12(a)), R_s_ is solution resistance; R_ct_ and CPE_dl_ respectively represent charge transfer resistance and electric double layer at the interface between Mg substrate and electrolyte solution, which are used to describe capacitance loop at high frequency. CPE_dl_ is constant phase element and replaces an ideal capacitor to account for the non-homogeneity in the system^48^. The CPE_dl_ is defined by two values of Y_0_ and n. The n value is the dispersion coefficient for CPE_dl_, which reflects the smoothness of the electrical double layer. If n = 1, CPE_dl_ is identical to a capacitor; If n = 0, CPE_dl_ represents a resistance; If n = 0.5, CPE_dl_ represents a Warburg impedance. R_L_ and L respectively represent resistance and inductance, which are used to describe the low frequency inductive loop, implying the initiation of localized corrosion^48^. For the pre-strained samples, the equivalent circuit model is illustrated in Fig. 12(b). In this model, R_s_ is the solution resistance; R_ct_ is charge transfer resistance; CPE_dl_ is electric double layer at the interface of electrolyte solution and Mg substrate.

Based on the proposed equivalent circuit models, the fitted data are listed in Table 2. It can be seen that for the as-rolled plate, the R_ct_ values of “ND”, “RD” and “TD” samples are 68.4, 146.6 and 198.9 Ωcm^2^, respectively. After being pre-strained, the R_ct_ values of “ND”, “RD” and “TD” samples are 1014.5, 983.9 and 1001.6 Ωcm^2^, respectively. Generally, the higher R_ct_ value implies the lower dissolution rate of Mg substrate^48^. Therefore, it demonstrates that the corrosion rates of the compressively strained samples are lower than that of the as-rolled samples. Meanwhile, the corrosion anisotropy between differently oriented samples was remarkably reduced. These results are consistent with the data measured from the polarization curves.

**Table 2:** Fitting results of the EIS for differently oriented samples before and after a 3% compressive strain along the rolling direction.

Condition		R_s_ (Ωcm^2^)	Y_0_ (μΩ^−1^ cm^−2^ s^n^)	n	R_ct_ (Ωcm^2^)	R_L_ (Ωcm^2^)	L (H cm^−2^)
As-rolled	“ND”	16.35 (±0.78)	52.9 (±8.5)	0.90 (±0.28)	68.4 (±5.7)	97.3 (±15.6)	28.39 (±8.35)
	“RD”	21.25 (±1.50)	18.2 (±2.1)	0.91 (±0.25)	146.6 (±10.5)	144.4 (±50.2)	17.81 (±4.52)
	“TD”	36.46 (±2.15)	13.6 (±1.5)	0.89 (±0.25)	198.9 (±15.3)	125.5 (±32.5)	28.67 (±6.68)
After a 3% compressive strain	“ND”	18.11 (±0.80)	20.5 (±2.0)	0.86 (±0.25)	1014.5 (±145.5)	—	—
	“RD”	23.84 (±1.68)	13.0 (±1.5)	0.90 (±0.27)	983.9 (±125.7)	—	—
	“TD”	33.97 (±2.25)	12.8 (±1.3)	0.93 (±0.28)	1001.6 (±138.6)	—	—

### Corrosion morphology after immersion testing

**Figure 13:**
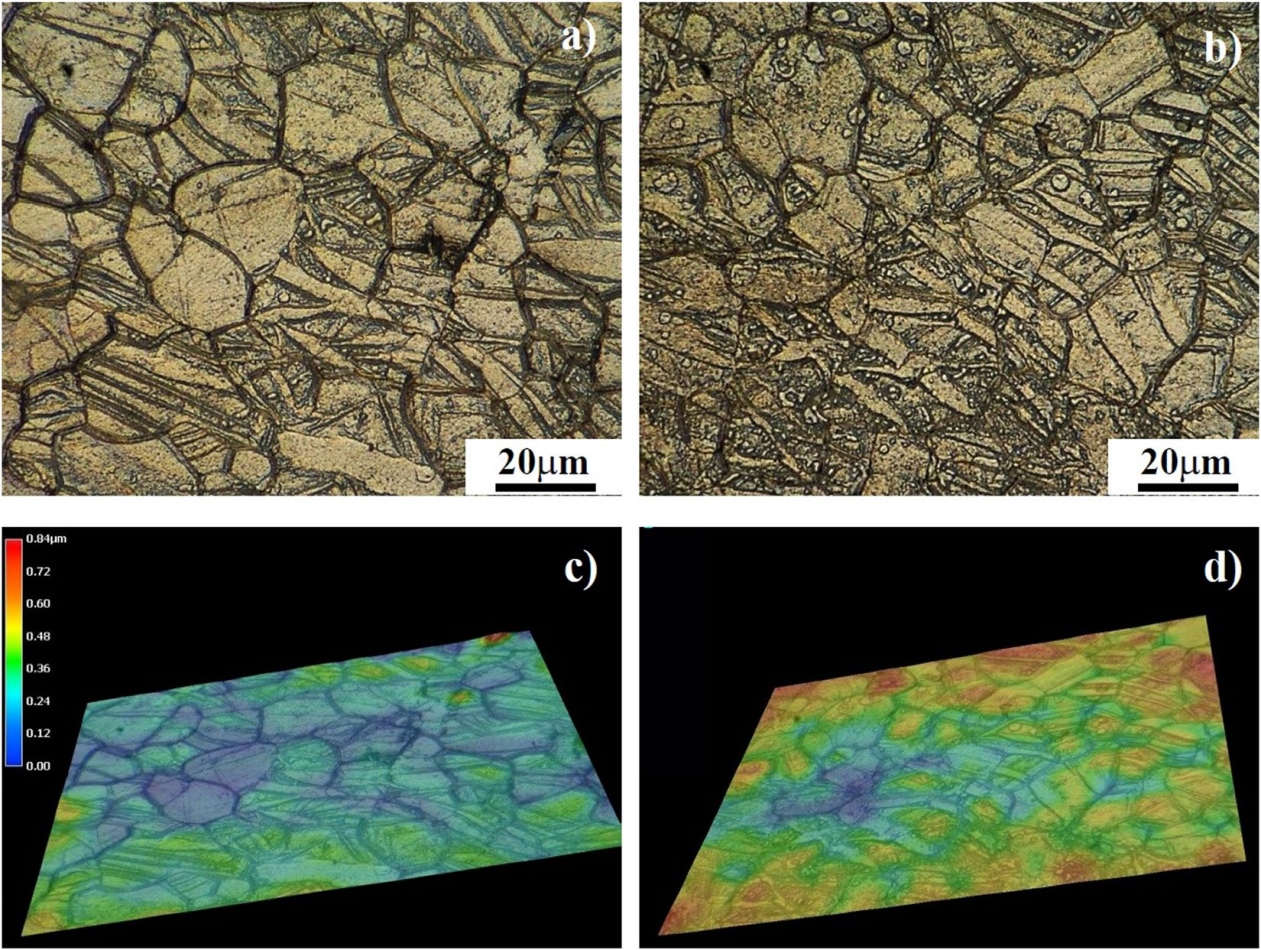
Optical observations to the surface of “ND” sample with a 3% compressive strain: (**a**) before and (**b**) after immersion in 0.9 wt.% NaCl solution at 37 °C for 60 min. Images (**c**) and (**d**) are the three dimensional images reflecting the micro change in surface evenness of samples in images (**a**) and (**b**), respectively.

To reflect the effect of twins on the corrosion behavior of the compressively strained samples, the surface morphologies of “ND” samples before and after being immersed in 0.9 wt.% NaCl solution at 37 °C for 60 min were selected for observation, as shown in Fig. 13.

Based on their three dimensional images (Fig. 13(c,d)), it can reflect the micro change in surface evenness of “ND” sample after immersion. For the polished surface, the surface evenness is good and the average height difference is about 0.4 μm. After immersion for 60 min, the surface evenness becomes much worse and its average value is 0.8 μm. Meanwhile, the height in the area with high densities of twins is much larger than the area with no or a small amount of twins. Since the height difference can directly reflect the corrosion severity of difference areas on sample surfaces, it proves that the matrix with no or less twins will be preferentially corroded and the activation of {10–12} twins can effectively improve the corrosion resistance of twinned area.

**Figure 14:**
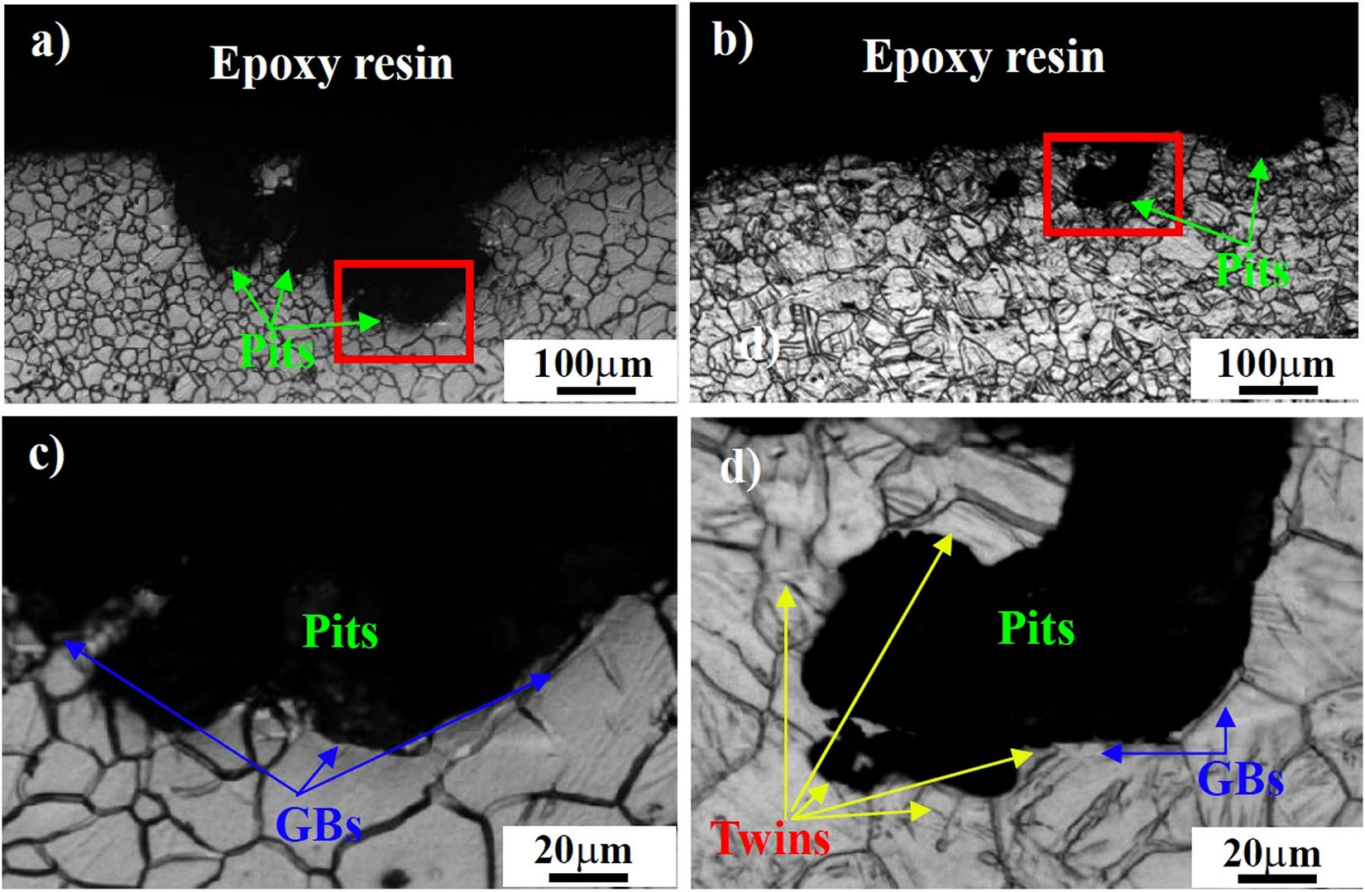
Cross-sectional morphologies of the corroded “ND” samples being immersed in 0.9 wt.% NaCl solution at 37 °C for 6 h: (**a**) before and (**b**) after a 3% compressive strain along the rolling direction.

Figure 14 shows the cross-sectional morphologies of corroded “ND” samples before and after a 3% compressive strain along the rolling direction. It can be seen that for the as-rolled condition, the surface of “ND” samples is severely corroded and the depth of pits can reach 300 μm (Fig. 14(a)). After being pre-strained, the corrosion attack on the surface of “ND” samples is obviously weakened and the depth of pits is less than 100 μm (Fig. 14(b)). High-magnified images reveal that grain and twin boundaries can act as physical barriers to the corrosion attack (Fig. 14(c,d)).

Generally, twin boundaries are fully coherent and have lower interfacial energies than those of the incoherent grain boundaries^49^. For pure magnesium, the {10–12} extension twin boundary energy is 0.11–0.20 J/m^2^ ^50^, which is far lower than grain boundary energy of 0.41–0.60 J/m^2^ ^51^. Therefore, with the thickening of surface films, twin boundaries can effectively suppress the degradation of films due to the inhomogeneous orientation stress^52^, which is confirmed by the EIS spectra (Fig. 11). Moreover, due to the activation of high densities of {10–12} twins in the pre-strained samples, the formed twin boundaries and original grain boundaries could remarkably increase the quantity of physical barriers to the corrosion attack. From the Fig. 9, it can be seen that the formation of filiform corrosion due to the growth of localized corrosion is quite easy for the non-strained samples. After being pre-strained, the growth and development of localized corrosion are effectively delayed or retarded. Therefore, it directly proves that the high densities of twin boundaries in Mg alloys can act as physical barriers for suppressing or delaying the corrosion attack. In addition, high densities of {10–12} twins could be simutaneously activated in differently oriented samples after a 3% compressive stain along the rolling direction of the plate. Thus, the beneficial effect of twin boundaries on weakening corrosion attack is the same for differently oriented surfaces. Following this, the corrosion anisotropy in as-rolled AZ31 Mg alloys with strong basal texture can be remarkably weakened due to the high densities of activated twins. Based on the description mentioned above, it demonstrates that the formation of high densities of {10–12} twins could be an effective way for simultaneously weakening the corrosion anisotropy and improving the corrosion resistance of wrought Mg alloys.

## Conclusions

Through investigating and comparing the corrosion behavior of the as-rolled AZ31 plate before and after a 3% compressive strain, the following conclusions can be drawn:

1. After a 3% compressive strain along the rolling direction of the plate, high densities of {10–12} twins can be activated in three differently oriented samples.
2. Due to the activation of high densities of {10–12} twins, the formed twin boundaries plus grain boundaries could act as physical barriers to corrosion attack, resulting in the simultaneous improvement of corrosion resistance of differently oriented samples.
3. The activation of {10–12} twins could weaken the occurrence of micro corrosion couples between the areas (different grains, twinned and untwinned Mg matrix) with different orientations, resulting in a very slight difference in the corrosion resistance between differently oriented samples.

## Methods

### Material preparation and treatments

The material investigated in this study was a commercially as-rolled AZ31 Mg alloy plate with a thickness of 20 mm and a rolling ratio of 5. The concrete chemical composition (in wt.%) is 3.12% Al, 1.16% Zn, 0.37% Mn and balance Mg. Cubic samples with dimension of 10 mm × 10 mm × 10 mm were cut from the plate. To remove the residual stress and avoid the overgrowth of grain size, annealing was carried out at 300 °C for 2 h in an air furnace followed by quenching in water. In magnesium, the theoretical maximum extension of 6.4% along the c-axis can be accommodated by the complete reorientation of {10–12} twins^53^. To heavily activate the {10–12} twins and avoid possible occurrence of micro cracks, some samples were performed a 3% compressive strain along the rolling direction of the plate after annealing. Then, samples were mounted with epoxy resin and the exposed surfaces have different orientation with respect to the plate. Among them, “ND”, “RD” and “TD” samples were defined as the surfaces with orientation parallel to the normal, rolling and transverse directions of the as-rolled plate, respectively.

### Microstructural analysis

Samples were ground with SiC papers progressively down to 2000 grit, and finely polished to 1 μm finish with ethanol lubricant. Then, the microstructures of samples were observed with an optical microscope (OM). The average grain sizes were determined by using the mean linear intercept method^54^. Samples for optical observation were etched with an etchant of (25 ml ethanol + 2 g picric acid + 5 ml acetic acid + 5 ml deionized water). The orientation maps of the grain structure and crystallographic textures were determined by electron backscatter diffraction (EBSD) using a scanning electron microscope (FEI NOVA 400) at an accelerating voltage of 20 kV, a step size of 1.5 μm, a sample tilt angle of 70° and a working distance of 15 mm. All the EBSD data were analyzed using the Channel 5 software. Samples for EBSD mapping were mechanically ground followed by electro-chemical polishing in an AC2 electrolyte solution at 20 V for 90 seconds. Macro texture analysis was performed on a D/Max 2400 X-ray diffractometer (XRD) using monochromatic Cu Kα radiation (wavelength: 0.154056 nm). Orientation distribution function (ODF) was calculated by MTEX software. Bunge notations of the Euler angles (φ_1_, Φ, φ_2_) were used to represent the ODF. The activated twins in the compressed samples were characterized by using transmission electron microscopy (FEI Tecnai G2 F20 TEM). The thin foils for TEM examination were mechanically ground and punched into discs of 3 mm in diameter. A twin-jet electro-polishing of the thin foils at −30 °C and 50 V was performed on a twin-jet electro-polisher (a Struers Tenupol-5) using a solution containing 3 vol.% perchloric acid in absolute ethanol. Finally, the foils were cleaned for 10 min by using a Gatan ion polishing system with an accelerating voltage of 3 kV and a temperature of −70 °C.

### Immersion testing

To reflect the severity of corrosion attack, samples with exposed surfaces of 10 mm × 10 mm were immersed in 0.9 wt.% NaCl solution at 37 °C for 0.5 h, 1 h and 2 h, respectively. The ratio of sample surface area (cm^2^) to the volume of NaCl solution (ml) was set to 1/50. Corrosion products after immersion were cleaned in a hot chromic acid bath consisting of 180 g/l CrO_3_. Afterwards, the morphologies of surfaces and cross-sections of immersed samples were observed using optical microscopy. Moreover, the micro thickness changes in twinned and untwinned areas on the pre-strained “ND” sample surface before and after immersion for 1 h were observed by using stereo optical microscope (Keyence VHX 2000). The principle of operation for obtaining 3D images with precise changes in height mainly contains two steps: 1) Automatically adjusting the focus and taking photos layer by layer at the same location; 2) Automatically combining these images.

### Hydrogen evolution testing

**Figure 15:**
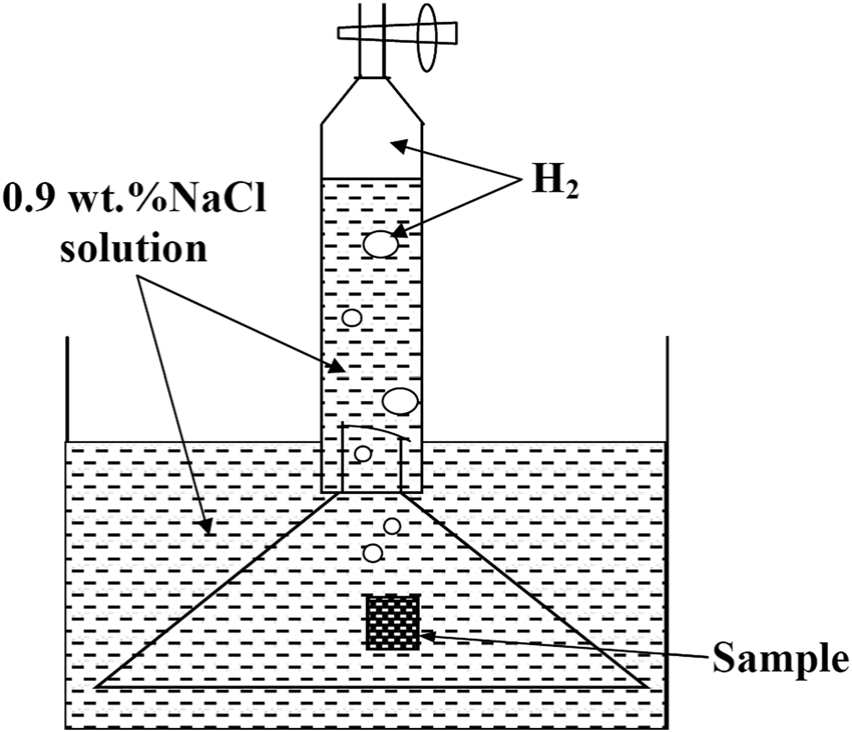
Schematic illustration of the apparatus for measuring the volume of evolved hydrogen.

For hydrogen evolution experiment, samples with an exposed area of 10 mm × 10 mm were immersed for up to 6 h at 37 °C and the evolved hydrogen bubbles were collected into a burette above the corroded samples. Schematic illustration of the apparatus for measuring the volume of evolved hydrogen is shown in Fig. 15. During immersion, no stirring or de-aerating was performed.

### Electrochemical testing

Potentiodynamic polarization experiments were carried out at a scan rate of 0.5 mV s^−1^ after being held at open-circuit potential (OCP) for 600 s using an EG&G potentiostat model 273 and a classical three electrode cell with Pt counter electrode and saturated calomel reference electrode. Samples were cold-mounted using epoxy resin, with an exposed area of 1 cm^2^. The polarization curves were fitted using CorrView software in the mode of Tafel (traditional). Using the same setup, electrochemical impedance spectroscopy (EIS) measurements were executed over a frequency range from 100 kHz to 10 mHz with 5 mV of amplitude of sinusoidal potential signals with respect to the OCP after 600 s stabilization in solution. The EIS spectra were fitted using the ZSimpWin 3.10 software. All electrochemical tests were repeated at least in triplicate to ensure the reproducibility.
